# Religiosity and Mental and Behavioural Health Among Community-Dwelling Middle-Aged and Older Adults in Thailand: Results of a Longitudinal National Survey in 2015–2020

**DOI:** 10.1007/s10943-025-02280-z

**Published:** 2025-02-25

**Authors:** Supa Pengpid, Karl Peltzer

**Affiliations:** 1 Department of Health Education and Behavioral Sciences, Faculty of Public Health, Mahidol University, Bangkok, Thailand; 2 Department of Public Health, Sefako Makgatho Health Sciences University, Pretoria, South Africa; 3 Department of Healthcare Administration, College of Medical and Health Science, Asia University, Taichung, Taiwan; 4 Department of Psychology, University of the Free State, Bloemfontein, South Africa; 5 Department of Psychology, College of Medical and Health Science, Asia University, Taichung, Taiwan

**Keywords:** Religious affiliation, Religious involvement, Mental health, Behavioural health older adults, Thailand

## Abstract

The aim of the study was to assess associations between religiousness (affiliation, and involvement) and five mental and five behavioural health indicators among middle-aged and older adults in a national longitudinal population survey in Thailand. The analytic sample consisted of 2863 participants, with two study assessments in 2015 and 2020. At baseline 91.5 percent were Buddhists and 8.2 percent were Muslims, and 42.6 percent a had high religious involvement. In the adjusted model, moderate and/or high religious involvement was negatively associated with four mental health and four behavioural health risk indicators. Furthermore, being a Buddhist was negatively associated with poor self-rated mental health status, depressive symptoms, insomnia symptoms and loneliness, and positively associated with alcohol use.

## Introduction

Thailand is experiencing rapid population ageing, thus increasing the burden of non-communicable diseases (NCDs), including mental and lifestyle disorders (Anantanasuwong, 2021; Kaufman et al., 2011; Prasartkul et al., 2018). Determinants of mental health include multiple individual, social and structural factors (Murniati et al., 2022; World Health Organization, 2022) and modifiable risk factors for NCDs include “tobacco use, physical inactivity, unhealthy diet and the harmful use of alcohol” (World Health Organization, 2023 p.1). Considering the importance of mental and lifestyle disorders, it is essential to identify its modifiable risk factors, such as religious involvement.

In Thailand, in 2021, 92.5 percent of the population were Buddhist, 5.4 percent Muslim, and 1.2 percent Christian (U.S. Department of State, 2022). Buddhist religion and health. A balanced mind–body relationship is the Buddhist definition of optimal health. When this equilibrium is upset, illness is said to manifest, and Buddhist activities are believed to strengthen and restore the unity of mind, body, and spirit (Weaver et al., 2008). According to Buddhist doctrine, good health is the outcome of deeds done well beginning the previous year, the previous second, or the previous life. Kamma law, however, is merely a subset of natural law. Therefore, the natural law also governs health and illness. This realization returns the responsibility for one's health to the person (Paonil & Sringernyuang, 2002). The Dhammapada-based core beliefs of traditional Buddhists and the religious practices that stem from them, like as mindfulness meditation, may have a positive impact on mental health and life satisfaction (Koenig, 2024). Buddhism also promotes moderation in consumption and mindful eating. The precept of not taking life and the mindfulness principle serve as the foundation for food practices in Buddhism. These rituals demonstrate Buddhism's dedication to kindness, restraint, and awareness in many facets of life (Arslan & Aydın, 2024).

To date, few studies have examined the relationship between religiosity and mental and behavioural health in the general adult or older adult population of Southeast Asia. For example, in Indonesian adults, lower religiosity increased the odds of depressive symptoms (Peltzer & Pengpid, 2018), and insomnia (Peltzer & Pengpid, 2019), and in Indian middle-aged and older adults, intrinsic and nonorganizational religiosity decreased the odds of depression (Pengpid & Peltzer, 2023). In the general adult population of Thailand, religious participation and being Buddhist decreased the odds of depressive symptoms (Xu et al., 2020), and in the older adult population of Thailand, religious participation increased cognitive functioning in men (Pothisiri &Vicerra, 2021). In an adult Muslim community in southern Thailand, village level of religious practice was protective against psychiatric symptoms (Ford et al., 2017), and a cross-sectional national study in Thailand showed that the formal application of Buddhist principles and the behavioral manifestation of Buddhist ideals are associated with greater levels of happiness (Winzer & Gray, 2019). Few local studies have shown the potential benefits of religion for mental health in Thailand (Ford et al., 2017; Pothisiri & Vicerra, 2021; Winzer & Gray, 2019; Xu et al., 2020). However, it appears no longitudinal national study on the impact of religiosity (affiliation and involvement) on a broad range of mental and behavioural health indicators has been conducted in Thailand, which prompted this study.

Past research into religion and mental and behavioural health has been conducted mainly in Western or Christian contexts, while non-Western, Buddhist or Muslim contexts remain underrepresented and may have different results (Hassan, 2015; Tey et al., 2018). As far as mental health is concerned, in a recent systematic review of cross-sectional and eight longitudinal studies (all longitudinal studies were not conducted in Southeast Asia) in people aged 60 and over, religious, and spiritual practice decreased the odds of poor mental health (anxiety and depressive symptoms, poor mental health status, and poor life satisfaction) (Coelho-Júnior et al., 2022). In three studies of adults in the United States, attendance of religious service reduced the odds of psychological distress (depression, anxiety, hopelessness, loneliness) and low life satisfaction (Chen et al., 2021). Five studies among adults (all conducted in USA), as reviewed in Hill et al. (2018), found that religious involvement was associated with better sleep quality. In the case of older adults in six lower resourced countries, religious minorities in Ghana and South Africa were more likely to suffer from depression (Fernández-Niño et al., 2019).

Regarding behavioural health, middle-aged and older Europeans who were more religious had lower odds of physical inactivity, alcohol consumption and smoking (Ahrenfeldt, et al., 2018). In Poland, religiousness was negatively associated with unhealthy behaviours (smoking and alcohol use) (Pawlikowski, et al., 2019), in the United States, religious service attendance lowered current smoking and heavy alcohol use (Chen et al., 2021) and in Denmark, religiosity was associated with a healthier diet (Svensson et al., 2020). In a study among older adults in USA, higher levels of religiosity were associated with higher use preventative health care services (Reindl Benjamins & Brown, 2004), and among adults in Texas, regular religious attendance was associated with more frequent use of preventive health exams (Hill et al., 2006).

As a result, the study was aimed at estimating the associations between religiousness (affiliation, and involvement) and five mental and five behavioural health indicators among middle-aged and older adults in a national longitudinal population survey in Thailand in 2015–2020.

## Methods

### Participants and Procedures

Analysis was done on two waves of health, aging, and retirement studies (HART) that were carried out in Thailand between 2015 and 2020. An adult (over 45) was randomly selected from each family and interviewed in the home as part of a multi-stage national sample plan; further information can be found here (Anantanasuwong et al., 2019). The study protocol was approved by the National Institute for Development Administration's Human Research Ethics Committee (ECNIDA 2020/00012), and participants gave their informed permission.

### Measures

#### Exposure Variables

*Religious affiliation* included, “What is your religion?” (Responses: Buddhist (n = 2619), Christian (n = 5), Muslim (n = 235), No religion (n = 1), and Other (n = 1), coded into Buddhist = 1 and Muslim and other = 0).

*Religious involvement* was measured with four items, (1) “Making merit and giving alms according to respected religious principles”, (2) “Prayer in the morning/before going to bed”, (3) “Performing merit-making activities at religious places according to the religions that the interviewees respect on important religious days,” and (4) “Observing important religious days that the interviewees respect.” The response alternatives for each question were "0 = never, 1 = rarely, 2 = often, and 3 = always." The item scores (range 0–12) were totaled and categorized as low (= 1:0–3), moderate (= 2:4–7), and high (= 3:8–12); Cronbach’s alpha was 0.83 (in 2015) and 0.74 (in 2020), respectively.

### Outcome Variables

#### Mental Health Outcomes

Mental health factors, including general mental health status, quality of life or happiness, depression, insomnia, and loneliness, were selected based on a previous review (Chen et al., 2021; Coelho-Júnior et al., 2022, Fernández-Niño et al., 2019; Ford et al., 2017; Peltzer & Pengpid, 2018, 2019; Pothisiri & Vicerra, 2021; Winzer & Gray, 2019; Xu et al., 2020).

"In general, how would you rate your mental health status?" was the question used to gauge self-reported mental health status. on a scale of 0 (extremely poor) to 10 (outstanding). A self-rated score of 0–7 (as opposed to 8–10) indicated poor mental health. Self-rated health measures consisting of just one item have been proven to be valid (Schnittker & Bacak, 2014).

"In general, how satisfied are you with your quality of life (or how happy do you feel)?" was the question used to gauge happiness and quality of life (QoL). on a scale of 0 (extremely poor) to 10 (outstanding). A self-rated score of 0–7 (as opposed to 8–10) indicated a low level of happiness or quality of life. It has been determined that single-item QoL assessments are valid (Zimmerman et al., 2006).

*Depressive symptoms* included 10 or more scores on the Center for Epidemiologic Studies Depression Scale (CES-D-10) (Andresen et al., 1994). Scores ≥ 10 demonstrated "sensitivity of 96.7% and specificity of 86.6% for depression" in an earlier study conducted among Thai adults (Nilmanut et al., 1997). In older community samples, such as those from Thailand, the CES-D has also been shown to be valid (Mackinnon et al., 1998). Cronbach’s alpha was 0.7 in this study (in 2015 and 2020).

The definition of insomnia symptoms was "having trouble falling asleep/insomnia in the past week," which was classified as “almost always (5–7 days) or often (3–4 days) as opposed to occasionally 1–2 days or extremely rarely/never.”

*Loneliness* was measured from the CES-D-10 item, “In the past week, how often did you experience feeling lonely?” defined as “almost always (5–7 days), often (3–4 days) or sometimes (1–2 days)” = 1 and “very rarely (less than one day) or none” = 0 (Andresen et al., 1994).

### Behavioural Health Indicators

Behavioural factors, including smoking, alcohol use, physical activity, dietary behaviour (meal skipping) and preventive health exams, were selected based on a previous review (Ahrenfeldt, et al., 2018; Chen et al., 2021; Pawlikowski, et al., 2019; Svensson et al., 2020).

*Tobacco smoking:* “Have you ever smoked cigarettes?” (“1 = yes, and still smoke now, 2 = yes, but quit smoking, and 3 = never”).

*Alcohol use:* “Have you ever drunk alcoholic beverages such as liquor, beer or wine?” (responses were: “1 = yes, and still drinking now, 2 = yes, but do not drink now, and 3 = never”).

*Physical exercise or activity* (frequency: “How often do you exercise?” (days per week) and duration of any type: “On the day you exercise, how long do you exercise?” (minutes), was grouped into “none = inactivity, 1–149 min/week = low activity, and ≥ 150 min/week = high activity in the past week.” (Kim, 2022).

*Meal skipping* was assessed with questions on “How many meals have you had in the last 2 days? Yesterday (breakfast, lunch, dinner; yes/no) and the day before yesterday (breakfast, lunch, dinner; yes/no)”. Meal skipping was “defined as skipping any breakfast, lunch, or dinner in the last two days” (Wild et al., 2023).

*Participation in annual health checks* is based on the question, "Have you undergone an annual health check-up last year?" (Yes/No).

### Covariates

*Sociodemographic variables* consisted of urban–rural residence, age groups (45–69 years and 70 years or more), sex (male/female), educational level (≤ elementary/ > elementary), marital status (widowed/non-widowed) and subjective economic status. Subjective economic status (“How satisfied are you with your economic situation?” was rated from 1 = lowest to 10 = highest, and low was defined as 1–5.

On a scale of 0 (extremely poor) to 10 (excellent), poor self-rated physical health status was classified as 0–6.0, with 7.0 serving as the median.

A modified Activities of Daily Living (ADL) scale consisting of four items (dressing, washing, eating, and bathing) was used to measure ADL disability (Katz et al., 1964). "0 = able to do it all by myself to 3 = need help for all steps" was one of the possible answers. One of the four components that cannot be completed alone is ADL disability. Cronbach’s α was 0.93 (in 2015) and 0.92 (in 2020).

#### Data Analysis

The percentage differences for the study year were determined using chi-square testing. To assess the longitudinal relationships between religiousness, mental and behavioural health indicators for the two study waves between 2015 and 2020, Generalized Estimating Equations Analysis (GEE), with the “logit” link function, was carried out. The first model is unadjusted, and the second model is adjusted for sociodemographic factors, ADL disability, physical health status, mental and behavioural health outcomes. Covariates were included based on earlier studies (AbdAleati et al., 2016; Ahrenfeldt et al., 2018; Chen et al., 2021; Pawlikowski et al., 2019), including activities of daily living and self-rated physical health (Ahrenfeldt et al., 2017). The results of the variable inflation factors (VIFs) statistics did not show collinearity. Only complete cases were analysed and *p* < 0.05 was considered significant. StataSE 16.0 (College Station, TX, USA) was used for statistical analysis.

## Results

The analytic sample consisted of 2863 participants in two study assessments in 2015 and 2020. At baseline, 91.5% were Buddhists and 8.2% were Muslims, 18.2% had no or low religious involvement and 42.6% had high religious involvement. The proportion of religious involvement was significantly higher among women than among men (*p* < 0.001) and decreased with age (*p* < 0.001). More than half of the participants (51.4%) lived in rural areas, 44.4% were men, and 15.7% had more than elementary education. Participants over the age of 70 increased from 36.6% in 2015 to 50.3% in 2020, and widows increased from 28.4% in 2015 to 32.8% in 2020. Significant differences occurred in subjective economic status, ADL disability, mental health factors (depressive symptoms, quality of life, mental health status, insomnia symptoms, and loneliness), and behavioural health factors (physical inactivity, meal skipping and participation past year health examination) (see Table 1).

**Table 1:** Descriptive statistics of the study variables over time, HART 2015–2020

Variables	Study year		*p*-value
	2015 (n = 2863)	2020 (n = 2863)	
	N (%)	N (%)	
Exposure variables			
Religious involvement Low Moderate High	522 (18.2) 1121 (39.2) 1220 (42.6)	664 (23.2) 1240 (43.3) 959 (33.5)	< 0.001
Religion (Buddhist)	2619 (91.5)	2585 (91.1)	0.895
Covariates			
Age (70 plus)	1040 (36.3)	1441 (50.3)	< 0.001
Sex (male)	1270 (44.4)		
Education (> elementary)	449 (15.7)		
Residence (rural)	1471 (51.4)		
Marital status (widowed)	802 (28.4)	937 (32.8)	0.002
Subjective economic status (low)	762 (27.7)	978 (35.7)	< 0.001
Poor self-rated physical health status	746 (26.6)	734 (25.6)	0.132
Functional disability	72 (2.6)	218 (7.6)	< 0.001
Mental health			
Self-reported poor mental health	798 (28.5)	685 (23.9)	< 0.001
Poor quality of life/happiness	769 (28.2)	983 (34.5)	< 0.001
Depressive symptoms	334 (12.8)	160 (5.6)	< 0.001
Insomnia symptoms	446 (15.7)	336 (11.7)	< 0.001
Loneliness	586 (20.8)	610 (21.3)	0.024
Behavioural health			
Current tobacco smoking	339 (11.9)	316 (11.0)	0.198
Current alcohol use	352 (12.4)	361 (12.6)	0.566
Physical inactivity	1606 (56.9)	1444 (50.5)	< 0.001
Meal skipping	156 (5.7)	379 (13.4)	< 0.001
No annual health check-up	1364 (47.6)	1142 (41.4)	< 0.001

Table 2 describes the frequency (never, rarely, often, and always) of the participation in four religious activities. “Always” was the highest for prayer (31.6%), followed by performing merit-making activities at religious places (29.0%), while “never” was the highest for observing important religious days (36.9%) and prayer (18.7%).

**Table 2:** Frequency distribution of individual religious activities of the pooled study sample, HART 2015–2020

Type of religious activity	Frequency of religious involvement			
	Never	Rarely	Often	Always
	%	%	%	%
Making merit and giving alms	16.9	33.0	24.6	25.4
Prayer	18.7	25.4	24.3	31.6
Performing merit-making activities at religious places	14.8	29.0	27.1	29.0
Observing important religious days	36.9	28.4	18.2	16.5

### Religiousness and Mental Health

In the adjusted model, high religious involvement was negatively associated with low quality of life (Adjusted Odds Ratio-AOR: 0.75, 95% Confidence Interval-CI 0.66–0.86, *p* < 0.001) poor mental health status (AOR: 0.71, 95% CI 0.61–0.83, *p* < 0.001), insomnia symptoms (AOR: 0.71, 95% CI 0.63–0.81, *p* < 0.001), and depressive symptoms (AOR: 0.63, 95% CI 0.63–0.81, *p* < 0.001). Furthermore, being Buddhist was negatively associated with loneliness (AOR: 0.53, 95% CI 0.44–0.64, *p* < 0.001), poor mental health status (AOR: 0.82, 95% CI 0.67–0.92, *p* < 0.001), depressive symptoms (AOR: 0.50, 95% CI 0.39–0.63, *p* < 0.001), and insomnia symptoms (AOR: 0.71, 95% CI 0.60–0.84, *p* < 0.001). In addition, in univariable analysis, moderate and/or high religious involvement was inversely associated with loneliness (see Table 3).

**Table 3:** Longitudinal associations between religious involvement and mental-ill health indicators

Outcome variables	Religious involvement	Model 1: unadjusted odds ratio (95% CI)	*p*-value	Model 2: adjusted odds ratio (95% CI)^a^	*p*-value
*Mental ill-health*					
Poor self-rated mental health status	Low Moderate High Buddhist	1 Reference 0.71 (0.62 to 0.80) 0.64 (0.56 to 0.73) 0.75 (0.63 to 0.89)	< 0.001 < 0.001 < 0.001	1 Reference 0.83 (0.71 to 0.95) 0.71 (0.61 to 0.83) 0.82 (0.67 to 0.92)	0.009 < 0.001 < 0.001
Study wave			2015 2020	1 Reference 0.62 (0.55 to 0.70)	< 0.001
Poor quality of life/happiness	Low Moderate High Buddhist	1 Reference 0.69 (0.61 to 0.71) 0.58 (0.51 to 0.66) 0.79 (0.67 to 0.95)	< 0.001 < 0.001 0.010	1 Reference 0.75 (0.66 to 0.86) 0.62 (0.54 to 0.71) 0.83 (0.68 to 1.00)	< 0.001 < 0.001 0.053
Study wave			2015 2020	1 Reference 1.00 (0.90 to 1.11)	0.954
Depressive symptoms	Low Moderate High Buddhist	1 Reference 0.75 (0.63 to 0.88) 0.51 (0.42 to 0.62) 0.46 (0.37 to 0.57)	< 0.001 < 0.001 < 0.001	1 Reference 0.83 (0.69 to 1.01) 0.63 (0.51 to 0.78) 0.50 (0.39 to 0.63)	0.065 < 0.001 < 0.001
Study wave			2015 2020	1 Reference 0.32 (0.26 to 0.39)	< 0.001
Insomnia symptoms	Low Moderate High Buddhist	1 Reference 1.04 (0.92 to 1.16) 0.72 (0.64 to 0.82) 0.67 (0.57 to 0.79)	0.560 < 0.001 < 0.001	1 Reference 1.04 (0.92 to 1.17) 0.71 (0.63 to 0.81) 0.71 (0.60 to 0.84)	0.545 < 0.001 < 0.001
Study wave			2015 2020	1 Reference 0.76 (0.69 to 0.83)	< 0.001
Loneliness	Low Moderate High Buddhist	1 Reference 1.01 (0.89 to 1.16) 0.72 (0.62 to 0.83) 0.51 (0.43 to 0.61)	0.867 < 0.001 < 0.001	1 Reference 1.11 (0.98 to 1.29) 0.91 (0.78 to 1.07) 0.53 (0.44 to 0.64)	0.089 0.274 < 0.001
			2015 2020	1 Reference 0.92 (0.82 to 1.04)	0.172

^a^Adjusted for age group, sex, education, marital status, subjective economic status, area of residence, Activities of Daily Living, self-rated physical health status, and all variables in the table

^***^p < 0.001; **p < 0.01; *p < 0.05; CI: Confidence Interval;

### Religiousness and Behavioural Health

In the adjusted model, moderate religious involvement was negatively associated with current tobacco smoking (AOR: 0.82, 95% CI 0.68–0.98, *p* = 0.033), and high religious involvement was negatively associated physical inactivity (AOR: 0.37, 95% CI 0.32–0.42, *p* < 0.001), meal skipping (AOR: 0.69, 95% CI 0.55–0.87, *p* < 0.001), and non-participation in past year health examination (AOR: 0.52, 95% CI 0.46–0.60, *p* < 0.001), Being Buddhist increased the odds of alcohol use (AOR: 27.15, 95% CI 7.92–93.03, *p* < 0.001). In addition, in univariable analysis, moderate and high religious involvement decreased the odds of current alcohol use (see Table 4).

**Table 4:** Longitudinal associations between religious involvement and behavioural health

Outcome variables	Religious involvement	Model 1: unadjusted odds ratio (95% CI)	*p*-value	Model 2: adjusted odds ratio (95% CI)^a^	*p*-value
*Behavioural health*					
Current tobacco smoking	Low	1 Reference		1 Reference	
	Moderate	0.72 (0.61 to 0.85)	< 0.001	0.82 (0.68 to 0.98)	0.033
	High	0.60 (0.50 to 0.71)	< 0.001	0.87 (0.71 to 1.06)	0.173
	Buddhist	0.96 (0.73 to 1.26)	0.754	0.96 (0.72 to 1.28)	0.783
Study wave			2015	1 Reference	0.713
			2020	0.97 (0.85 to 1.12)	
Current alcohol use	Low	1 Reference		1 Reference	
	Moderate	0.77 (0.66 to 0.91)	0.002	0.88 (0.73 to 1.06)	0.163
	High	0.60 (0.50 to 0.71)	< 0.001	0.86 (0.70 to 1.06)	0.160
	Buddhist	16.21 (6.38 to 41.16)	< 0.001	27.15 (7.92 to 93.03)	< 0.001
			2015	1 Reference	< 0.001
			2020	1.34 (1.15 to 1.55)	
Physical inactivity	Low	1 Reference		1 Reference	
	Moderate	0.50 (0.44 to 0.57)	< 0.001	0.53 (0.46 to 0.60)	< 0.001
	High	0.36 (0.32 to 0.41)	< 0.001	0.37 (0.32 to 0.42)	< 0.001
	Buddhist	0.89 (0.75 to 1.05)	0.170	0.90 (0.75 to 1.08)	0.246
Study wave			2015	1 Reference	< 0.001
			2020	0.60 (0.55 to 0.66)	
Meal skipping	Low	1 Reference		1 Reference	
	Moderate	0.98 (0.80 to 1.19)	0.824	0.93 (0.75 to 1.14)	0.476
	High	0.69 (0.56 to 0.86)	< 0.001	0.69 (0.55 to 0.87)	< 0.001
	Buddhist	0.92 (0.69 to 1.24)	0.596	0.94 (0.69 to 1.28)	0.694
			2015	1 Reference	< 0.001
			2020	2.46 (2.09 to 1.80)	
No annual health check-up	Low	1 Reference		1 Reference	
	Moderate	0.60 (0.53 to 0.67)	< 0.001	0.60 (0.53 to 0.68)	< 0.001
	High	0.51 (0.45 to 0.58)	< 0.001	0.52 (0.46 to 0.60)	< 0.001
	Buddhist	1.12 (0.95 to 1.33)	0.188	1.07 (0.89 to 1.28)	0.482
Study wave			2015	1 Reference	< 0.001
			2020	0.67 (0.61 to 0.74)	

^a^Adjusted for age group, sex, education, marital status, subjective economic status, area of residence, Activities of Daily Living, self-rated physical health status, and all variables in the table

^***^p < 0.001; **p < 0.01; *p < 0.05; CI: Confidence Interval;

## Discussion

The study was designed to evaluate for the first time the association between religiosity (affiliation, and involvement) and a broad range of mental and behavioural health indicators in a longitudinal national sample of community-dwelling ageing adults in Thailand in 2015–2020. Religious affiliation was almost 100% of this middle-aged and older adult population in Thailand, similar to almost 100% of previous national data in Thailand (U.S. Department of State (2022). As with the previous Thai national data (U.S. Department of State, 2022), the proportion of Buddhists (92.5%), Muslims (5.4%) and Christians (1.2%) was slightly similar in this survey (91.5% Buddhists, 8.2% Muslims and 0.2% Christians). Consistent with previous research (Santero et al., 2019), we found that women had higher religious involvement than men. Furthermore, in our study, religious involvement (nonorganizational and organizational religiosity) declined with age, which is consistent with middle-aged and older adults in India (Pengpid & Peltzer, 2023).

As for the results of mental health, this study showed that moderate and/or high religious involvement was negatively associated depressive and insomnia symptoms, low quality of life or happiness and poor mental health status. These findings are consistent with previous reviews and studies, mainly from Western or Christian contexts (Chen et al., 2021; Coelho-Júnior et al., 2022; Hill et al., 2018; Winzer & Gray, 2019), expanding our knowledge to non-Western, Buddhist or Muslim contexts. The mechanism for reducing poor mental health by religious participation can be explained by stress adaptation models (Koenig, 2018), prayer and nonorganized religious activities can help reduce or cope with life stress, thus reducing mental symptoms (Reyes-Ortiz, 2020). In terms of loneliness, religious involvement may protect against loneliness in later life by incorporating senior citizens into more extensive and encouraging social networks (Rote et al., 2013). Regarding insomnia symptoms, by reducing the mental, chemical, and physiological arousal linked to substance abuse, stress exposure, and allostatic load, religious participation may be linked to better sleep outcomes (Hill et al., 2018). Furthermore, this study showed that being Buddhist was negatively associated with loneliness, poor self-rated mental health status, depressive and insomnia symptoms. This result seems to confirm previous research (Fernández-Niño et al., 2019; Xu et al., 2020) that religious minorities, in this case Muslims in Thailand, are more vulnerable to poor mental health.

Regarding behavioural health outcomes, this study found that moderate and/or high religious involvement was negatively associated with current tobacco smoking, physical inactivity, meal skipping and non-participation in past year health examination. These findings are in line with some previous studies from Western or Christian contexts (Ahrenfeldt, et al., 2018; Chen et al., 2021; Hill et al., 2006; Reindl Benjamins & Brown, 2004; Svensson et al., 2020) showing an association between religiousness and lower odds of substance use, physical inactivity, and unhealthy diet, and higher odds of attending preventive health exams, expanding our knowledge to non-Western, Buddhist or Muslim contexts.

In a study among adult smokers of Thai Buddhists and Malaysian Muslims, 79% and 88%, respectively, believed that their religion discourages smoking (Yong et al., 2009). People's religious convictions may inspire them to take annual medical check-ups and live healthier lives. Furthermore, by offering knowledge, practical assistance, or the real preventative care services, religion may make it possible for people to utilize these kinds of services (Reindl Benjamins & Brown, 2004). While some studies (Ahrenfeldt, et al., 2018; Chen et al., 2021; Pawlikowski, et al., 2019) found a negative association between religious participation and alcohol use, we found this association only in univariable analysis. Furthermore, this study showed that being Buddhist was positively associated with alcohol use. In a systematic review, it was found that “religious affiliations, such as Buddhism, Catholicism and Lutheranism, appear to be risk factors for alcohol consumption” (Chagas et al., 2023, p.238). Overall, exposure to religious doctrines that discourage particular health-relevant behaviours (e.g., Buddhism advocates moderation in consumption and mindful eating) may result in healthier lifestyles. Certain religious prohibitions may help explain why religious people might abstain from certain health-related behaviours (such as Muslims abstaining from alcohol), but they are unable to explain how religious participation affects health-related behaviours that are not specifically mentioned in religious texts (such as smoking and exercise). Nonetheless, it is plausible that religious communities follow broad theological precepts regarding the instrumental significance of physical well-being as a pathway to increased spiritual engagement and dedication (Hill et al., 2007).

### Study Limitations

Study measures were assessed by self-report, which may have biased responses. Although the study adjusted for a wide range of covariates, we cannot rule out reverse causality. The measure of religiousness included nonorganizational and organizational religiosity but did not assess intrinsic religiosity.

## Conclusions

A high religious involvement was observed among middle-aged and older adults in Thailand. Religious participation reduced the likelihood of symptoms of depression and insomnia, poor quality of life or happiness, poor mental health, current smoking, physical inactivity, meal skipping and non-participation to the annual health check-up. Furthermore, being a Buddhist was negatively associated with loneliness, poor mental health, depression and insomnia symptoms, and being a Buddhist increased the odds of alcohol consumption. Compared to Muslims, Buddhists have lower probability of poor mental health and higher probability of alcohol use. Higher religious participation was negatively associated with four mental health indicators and four health-related risk behaviours. Health providers may consider the potential benefits of religion to reduce poor mental and behavioural problems among middle-aged and elderly adults in Thailand.

## Data Availability

Data is publicly available at Health, Aging, and Retirement in Thailand (HART): https://hart.nida.ac.th/download-center/

## References

[CR1] AbdAleati, N. S., Mohd Zaharim, N., & Mydin, Y. O. (2016). Religiousness and mental health: Systematic review study. *Journal of Religion and Health,**55*(6), 1929–1937. 10.1007/s10943-014-9896-1 27654836 10.1007/s10943-014-9896-1

[CR2] Ahrenfeldt, L. J., Möller, S., Andersen-Ranberg, K., Vitved, A. R., Lindahl-Jacobsen, R., & Hvidt, N. C. (2017). Religiousness and health in Europe. *European Journal of Epidemiology,**32*(10), 921–929. 10.1007/s10654-017-0296-1 28840406 10.1007/s10654-017-0296-1

[CR3] Ahrenfeldt, L. J., Möller, S., Hvidt, N. C., & Lindahl-Jacobsen, R. (2018). Religiousness and lifestyle among Europeans in SHARE. *Public Health,**165*, 74–81. 10.1016/j.puhe.2018.09.009 30384031 10.1016/j.puhe.2018.09.009

[CR5] Anantanasuwong, D. (2021). Population ageing in Thailand: Critical issues in the twenty first century. In P. Narot & N. Kiettikunwong (Eds.), *Education for the elderly in the Asia Pacific (Education in the Asia-Pacific region: Issues concerns and prospects)* (59th ed., pp. 31–56). Springer.

[CR6] Anantanasuwong, D., Theerawanviwat, D., & Siripanich, P. (2019). Panel survey and study on health, aging, and retirement in Thailand. In D. Gu & M. Dupre (Eds.), *Encyclopedia of gerontology and population aging. *Springer. 10.1007/978-3-319-69892-2_982-1

[CR7] Andresen, E. M., Malmgren, J. A., Carter, W. B., & Patrick, D. L. (1994). Screening for depression in well older adults: evaluation of a short form of the CES-D (Center for epidemiologic studies depression scale). *American Journal of Preventive Medicine,**10*, 77–84. 8037935

[CR8] Arslan, S., & Aydın, A. (2024). Religious dietary practices: Health outcomes and psychological insights from various countries. *Journal of Religion and Health,**63*(5), 3256–3273. 10.1007/s10943-024-02110-8 39154120 10.1007/s10943-024-02110-8

[CR9] Chagas, C., Martins, L. B., Bezerra, A. G., Paula, T. C. S., Xavier, A. C. A., Zangari, W., & Galduróz, J. C. F. (2023). A systematic review on alcohol consumption among non-religious and religious adults. *Substance Use & Misuse,**58*(2), 238–256. 10.1080/10826084.2022.2155477 36510842 10.1080/10826084.2022.2155477

[CR10] Chen, Y., Kim, E. S., & VanderWeele, T. J. (2021). Religious-service attendance and subsequent health and well-being throughout adulthood: Evidence from three prospective cohorts. *International Journal of Epidemiology,**49*(6), 2030–2040. 10.1093/ije/dyaa120 32793951 10.1093/ije/dyaa120 PMC7825951

[CR11] Coelho-Júnior, H. J., Calvani, R., Panza, F., Allegri, R. F., Picca, A., Marzetti, E., & Alves, V. P. (2022). Religiosity/spirituality and mental health in older adults: A systematic review and meta-analysis of observational studies. *Frontiers in Medicine,**9*, 877213. 10.3389/fmed.2022.877213 35646998 10.3389/fmed.2022.877213 PMC9133607

[CR12] Fernández-Niño, J. A., Bojorquez, I., Becerra-Arias, C., & Astudillo-Garcia, C. I. (2019). Religious affiliation and major depressive episode in older adults: A cross-sectional study in six low- and middle- income countries. *BMC Public Health,**19*(1), 460. 10.1186/s12889-019-6806-1 31039777 10.1186/s12889-019-6806-1 PMC6492427

[CR13] Ford, K., Jampaklay, A., & Chamratrithirong, A. (2017). Mental health in a conflict area: Migration, economic stress and religiosity in the three southernmost provinces of Thailand. *The International Journal of Social Psychiatry,**63*(2), 91–98. 10.1177/0020764016685119 28024446 10.1177/0020764016685119

[CR14] Hassan, S. H. (2015). Effects of religious behavior on health-related lifestyles of Muslims in Malaysia. *Journal of Religion and Health,**54*(4), 1238–1248. 10.1007/s10943-014-9861-z 24729099 10.1007/s10943-014-9861-z

[CR15] Hill, T. D., Burdette, A. M., Ellison, C. G., & Musick, M. A. (2006). Religious attendance and the health behaviors of Texas adults. *Preventive Medicine,**42*(4), 309–312. 10.1016/j.ypmed.2005.12.005 16445971 10.1016/j.ypmed.2005.12.005

[CR16] Hill, T. D., Deangelis, R., & Ellison, C. G. (2018). Religious involvement as a social determinant of sleep: An initial review and conceptual model. *Sleep Health,**4*(4), 325–330. 10.1016/j.sleh.2018.04.001 30031524 10.1016/j.sleh.2018.04.001

[CR17] Hill, T. D., Ellison, C. G., Burdette, A. M., & Musick, M. A. (2007). Religious involvement and healthy lifestyles: Evidence from the survey of Texas adults. *Annals of Behavioral Medicine: A Publication of the Society of Behavioral Medicine,**34*(2), 217–222. 10.1007/BF02872676 17927560 10.1007/BF02872676

[CR18] Katz, S., Ford, A. B., Heiple, K. G., & Newill, V. A. (1964). Studies of illness in the aged: Recovery after fracture of the hip. *Journal of Gerontology,**19*, 285–293. 10.1093/geronj/19.3.285 14179683 10.1093/geronj/19.3.285

[CR19] Kaufman, N. D., Chasombat, S., Tanomsingh, S., Rajataramya, B., & Potempa, K. (2011). Public health in Thailand: Emerging focus on non-communicable diseases. *The International Journal of Health Planning and Management,**26*(3), e197–e212. 10.1002/hpm.1078 21796679 10.1002/hpm.1078 PMC8780404

[CR20] Kim, J. H. (2022). Regular physical exercise and its association with depression: A population-based study short title: Exercise and depression. *Psychiatry Research,**309*, 114406. 10.1016/j.psychres.2022.114406 35074644 10.1016/j.psychres.2022.114406

[CR21] Koenig, H. G. (2018). *Religion and mental health: Research and clinical applications*. Academic Press (Elsevier).

[CR22] Koenig, H. G. (2024). Mental Health and Well-Being in Buddhism. In H. S. Moffic, R. R. Gogineni, J. R. Peteet, N. K. Aggarwal, N. K. Malhi, & A. Hankir (Eds.), *Eastern Religions, Spirituality, and Psychiatry. *Springer. 10.1007/978-3-031-56744-5_11

[CR23] Mackinnon, A., McCallum, J., Andrews, G., & Anderson, I. (1998). The center for epidemiological studies depression scale in older community samples in Indonesia, North Korea, Myanmar, Sri Lanka, and Thailand. *The Journals of Gerontology. Series B, Psychological Sciences and Social sciences,**53*(6), P343–P352. 10.1093/geronb/53b.6.p343 9826965 10.1093/geronb/53b.6.p343

[CR24] Murniati, N., Al Aufa, B., Kusuma, D., & Kamso, S. (2022). A scoping review on biopsychosocial predictors of mental health among older adults. *International Journal of Environmental Research and Public Health,**19*(17), 10909. 10.3390/ijerph191710909 36078627 10.3390/ijerph191710909 PMC9518331

[CR25] Nilmanut, S., Kuptniratsaikul, V., Pekuman, P., & Tosayanonda, O. (1997). The study of the center for epidemiologic studies-depression scale (CES-D) in Thai people Siriraj hospital. *ASEAN Journal of Rehabilitation Medicine,**6*(3), 25–29.

[CR26] Paonil, W., & Sringernyuang, L. (2002). Buddhist perspectives on health and healing. *The Chulalongkorn Journal of Buddhist Studies,**1*(2), 93–104.

[CR27] Pawlikowski, J., Białowolski, P., Węziak-Białowolska, D., & VanderWeele, T. J. (2019). Religious service attendance, health behaviors and well-being-an outcome-wide longitudinal analysis. *European Journal of Public Health,**29*(6), 1177–1183. 10.1093/eurpub/ckz075 31038170 10.1093/eurpub/ckz075

[CR28] Peltzer, K., & Pengpid, S. (2018). High prevalence of depressive symptoms in a national sample of adults in Indonesia: Childhood adversity, sociodemographic factors and health risk behaviour. *Asian Journal of Psychiatry,**33*, 52–59. 10.1016/j.ajp.2018.03.017 29529418 10.1016/j.ajp.2018.03.017

[CR29] Peltzer, K., & Pengpid, S. (2019). Prevalence, social and health correlates of insomnia among persons 15 years and older in Indonesia. *Psychology, Health & Medicine,**24*(6), 757–768. 10.1080/13548506.2019.1566621 10.1080/13548506.2019.1566621 30618274

[CR30] Pengpid, S., & Peltzer, K. (2023). Religiosity and depression among community-dwelling older adults in India: Results of a national survey in 2017–2018. *Journal of Religion and Health,**62*(5), 3006–3016. 10.1007/s10943-022-01640-3 36006530 10.1007/s10943-022-01640-3

[CR31] Pothisiri, W., & Vicerra, P. M. (2021). Cognitive function, co-residence, and social participation among older persons in Thailand. *The Social Science Journal*. 10.1080/03623319.2020.1851076

[CR32] Prasartkul, P., Thaweesit, S., & Chuanwan, S. (2018). Prospects and contexts of demographic transitions in Thailand. *Journal of Population and Social Studies,**27*(1), 1–22.

[CR33] Reindl Benjamins, M., & Brown, C. (2004). Religion and preventative health care utilization among the elderly. *Social Science & Medicine,**58*(1), 109–118. 10.1016/s0277-9536(03)00152-7 14572925 10.1016/s0277-9536(03)00152-7

[CR350] Reyes-Ortiz, C. A., Payan, C., Altamar, G., Montes, J. F. G., & Koenig, H. G. (2020). Religiosity and depressive symptoms among older adults in Colombia.* Aging & mental health*, *24*(11), 1879–1885. 10.1080/13607863.2019.1660851 10.1080/13607863.2019.1660851 33076684

[CR34] Rote, S., Hill, T. D., & Ellison, C. G. (2013). Religious attendance and loneliness in later life. *The Gerontologist,**53*(1), 39–50. 10.1093/geront/gns063 22555887 10.1093/geront/gns063 PMC3551208

[CR35] Santero, M., Daray, F. M., Prado, C., Hernández-Vásquez, A., & Irazola, V. (2019). Association between religiosity and depression varies with age and sex among adults in South America: Evidence from the CESCAS I study. *PLoS ONE,**14*(12), e0226622. 10.1371/journal.pone.0226622 31841570 10.1371/journal.pone.0226622 PMC6913958

[CR36] Schnittker, J., & Bacak, V. (2014). The increasing predictive validity of self-rated health. *PLoS ONE,**9*(1), e84933. 10.1371/journal.pone.0084933 24465452 10.1371/journal.pone.0084933 PMC3899056

[CR37] Svensson, N. H., Hvidt, N. C., Nissen, S. P., Storsveen, M. M., Hvidt, E. A., Søndergaard, J., & Thilsing, T. (2020). Religiosity and health-related risk behaviours in a secular culture-is there a correlation? *Journal of Religion and Health,**59*(5), 2381–2396. 10.1007/s10943-019-00919-2 31562592 10.1007/s10943-019-00919-2 PMC7502034

[CR38] Tey, S. E., Park, M. S., & Golden, K. J. (2018). Religiosity and healthy lifestyle behaviours in Malaysian Muslims: The mediating role of subjective well-being and self-regulation. *Journal of Religion and Health,**57*(6), 2050–2065. 10.1007/s10943-017-0420-2 28647911 10.1007/s10943-017-0420-2

[CR39] U.S. Department of State (2022). 2022 Report on international religious freedom: Thailand. URL: https://www.state.gov/wp-content/uploads/2023/05/441219-THAILAND-2022-INTERNATIONAL-RELIGIOUS-FREEDOM-REPORT.pdf (accessed 10 Nov 2023)

[CR40] Weaver, A. J., Vane, A., & Flannelly, K. J. (2008). A review of research on Buddhism and health: 1980–2003. *Journal of Health Care Chaplaincy,**14*(2), 118–132. 10.1080/08854720802129075 18697355 10.1080/08854720802129075

[CR41] Wild, H., Baek, Y., Shah, S., Gasevic, D., & Owen, A. (2023). The socioecological correlates of meal skipping in community-dwelling older adults: A systematic review. *Nutrition Reviews,**81*(2), 168–179. 10.1093/nutrit/nuac050 35913413 10.1093/nutrit/nuac050

[CR42] Winzer, L., & Gray, R. S. (2019). The role of Buddhist practices in happiness and health in Thailand: A structural equation model. *Journal of Happiness Studies,**20*, 411–425. 10.1007/s10902-017-9953-z

[CR43] World Health Organization (2022). Mental health. URL: https://www.who.int/news-room/fact-sheets/detail/mental-health-strengthening-our-response (accessed 10 Nov 2023)

[CR44] World Health Organization (2023). Noncommunicable diseases. URL: https://www.who.int/news-room/fact-sheets/detail/noncommunicable-diseases (accessed 10 Nov 2023)

[CR45] Xu, T., Xu, X., Sunil, T., & Sirisunyaluck, B. (2020). Buddhism and depressive symptoms among married women in urban Thailand. *International Journal of Environmental Research and Public Health,**17*(3), 761. 10.3390/ijerph17030761 31991732 10.3390/ijerph17030761 PMC7037506

[CR46] Yong, H. H., Hamann, S. L., Borland, R., Fong, G. T., & Omar, M. (2009). Adult smokers’ perception of the role of religion and religious leadership on smoking and association with quitting: a comparison between Thai Buddhists and Malaysian Muslims. *Social Science & Medicine,**69*(7), 1025–1031. 10.1016/j.socscimed.2009.07.042 19695758 10.1016/j.socscimed.2009.07.042 PMC2775092

[CR47] Zimmerman, M., Ruggero, C. J., Chelminski, I., Young, D., Posternak, M. A., Friedman, M., Boerescu, D., & Attiullah, N. (2006). Developing brief scales for use in clinical practice: The reliability and validity of single-item self-report measures of depression symptom severity, psychosocial impairment due to depression, and quality of life. *The Journal of Clinical Psychiatry,**67*(10), 1536–1541. 10.4088/jcp.v67n1007 17107244 10.4088/jcp.v67n1007

